# CSF1R T567M mutation induces microglial dysfunction and synaptic impairment in patient iPSC-derived cerebral organoids of CSF1R-related disorder

**DOI:** 10.1038/s41420-026-02995-2

**Published:** 2026-03-12

**Authors:** Li Chi, Haitao Tu, Zhihong Li, Lifeng Qiu, Zhi-Wei Zhang, Sook-Yoong Chia, Jayne Yi Tan, Ivy A. W. Ho, Yuin-Han Loh, Eng-King Tan, Wei Teng, Zhong Pei, Zbigniew K. Wszolek, Adeline S. L. Ng, Li Zeng

**Affiliations:** 1 Guangdong Provincial Key Laboratory of Stomatology, Hospital of Stomatology, Institute of Stomatological Research, Guanghua School of Stomatology, Sun Yat-sen University, Guangzhou, China; 2 Neural Stem Cell Research Lab, Research Department, National Neuroscience Institute, Singapore, Singapore; 3 Neuroscience & Behavioral Disorders Program, DUKE-NUS Graduate Medical School, Singapore, Singapore; 4 Department of Neurology, National Neuroscience Institute, Singapore, Singapore; 5 Molecular Neurotherapeutics Laboratory, National Neuroscience Institute, Singapore, Singapore; 6 Department of Physiology, Yong Loo Lin School of Medicine, National University of Singapore, Singapore, Singapore; 7 Duke-NUS Medical School, Singapore, Singapore; 8 Institute of Molecular and Cell Biology (IMCB), A*STAR (Agency for Science, Technology and Research), Singapore, Singapore; 9 Research Department, National Neuroscience Institute, Singapore General Hospital (SGH) Campus, Singapore, Singapore; 10 Guangdong Provincial Key Laboratory of Diagnosis and Treatment of Major Neurological Diseases, Department of Neurology, The First Affiliated Hospital; National Key Clinical Department and Key Discipline of Neurology, Sun Yat-Sen University, Guangzhou, China; 11 Department of Neurology, Mayo Clinic Florida, Jacksonville, FL USA; 12 Centre for Molecular Neuropathology, Lee Kong Chian School of Medicine, Nanyang Technological University, Singapore, Singapore

**Keywords:** Neurogenesis, Neurodegeneration, Immunopathogenesis, Stem-cell research

## Abstract

CSF1R-related disorder (CSF1R-RD) is a rare autosomal dominant neurodegenerative disease characterized by cognitive decline, motor dysfunction, psychiatric symptoms, and white matter abnormalities. It is caused by mutations in the *CSF1R* gene. Despite the identification of many pathogenic CSF1R variants, the molecular mechanisms behind neuropathogenesis in CSF1R-RD remain poorly understood due to the lack of disease modeling. This study focuses on a novel CSF1R mutation, T567M, located outside the tyrosine kinase domain, whose pathogenic impact has not been characterized. To gain molecular insights into the pathogenic mechanisms of the CSF1R-T567M mutation, we established an induced pluripotent stem cell (iPSC) model system consisting of mutant (CSF1R-MT) and CRISPR/Cas9-corrected isogenic control lines. Using these iPSCs, we generated iPSC-derived microglia (iMGL) and cerebral organoids (COs). Through RNA sequencing, we identified altered genes and pathways involved in neuroinflammation in MT iMGL. We then investigated microglial migration, phagocytosis, cytokine profiling, neurodevelopment, and synaptic function in iMGL and iMGL-CO co-culture to study the role of T567M mutation in CSF1R-RD. Our research revealed that the CSF1R-MT caused haploinsufficiency of CSF1R, reducing autophosphorylation of CSF1R at Tyr546 and activating autophagy. CSF1R-MT iMGL induced neuroinflammation, increased phagocytosis, and impaired migration. Transcriptomic analysis showed upregulation of immune activity and downregulation of synaptic function. Additionally, CSF1R-MT promoted proliferation, inhibited neural differentiation and maturation, and caused neurodevelopmental defects in COs. Whole-cell patch-clamp recordings indicated impaired synaptic function in CSF1R-MT COs. Furthermore, CSF1R-MT microglia impaired synaptic protein expression when co-cultured with CSF1R-MT COs. Collectively, our study provides detailed mechanistic insights into the pathogenesis driven by the CSF1R-T567M mutation, highlighting the critical role of CSF1R signaling in neural homeostasis. This isogenic iPSC model serves as a valuable platform for probing mutation-specific mechanisms and future therapeutic screening.

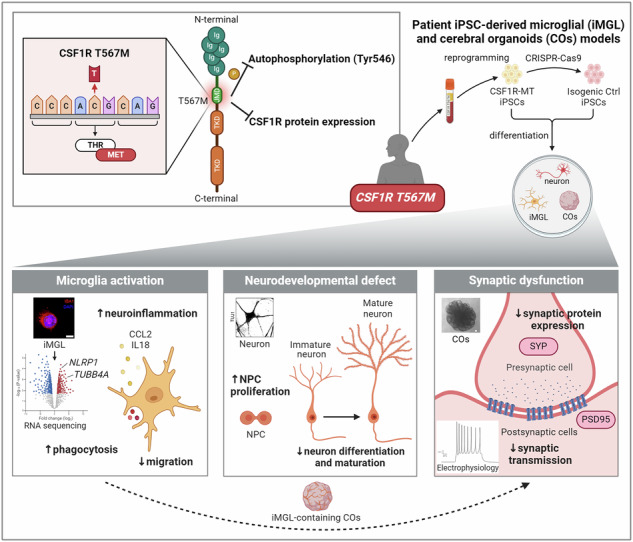

## Introduction

CSF1R-related disorder (CSF1R-RD) is a rare and progressive neurodegenerative disease that is closely related to Adult-onset Leukoencephalopathy with Axonal Spheroids and Pigmented Glia (ALSP). It is also called Hereditary Diffuse Leukoencephalopathy with Spheroids (HDLS) and is characterized by mutations in the colony-stimulating factor-1 receptor (*CSF1R*) gene [1–4]. AARS/AARS1 (alanyl-tRNA synthetase)-related leukoencephalopathy (HDLS2) is genetically distinct from CSF1R-RD, although the two disorders share similar clinical, radiological, and pathological features [3, 5]. Similar to many neurodegenerative conditions, CSF1R-RD is currently incurable. There is no clear standard treatment protocol for ALSP, and the disease progression is often relentless. Most current interventions are palliative and aimed at alleviating symptoms, with limited effects on halting the underlying pathology. Hematopoietic stem cell transplantation, which aims to replace the diseased microglial population, is currently the most investigated therapeutic approach and has shown promise in some cases [6]. However, its overall efficacy and optimal timing are still under investigation [3, 7]. Therefore, elucidating the molecular pathogenesis of CSF1R-RD remains critical for developing definitive treatments.

The underlying cause of CSF1R-RD lies in loss-of-function mutations within the *CSF1R* gene, located on human chromosome 5q32. This gene spans 22 exons and encodes a class of tyrosine kinase receptors [8]. Despite extensive research, the precise functional implications of all known CSF1R mutations and the complete genetic landscape contributing to CSF1R encephalopathy remain unknown [9]. Globally, more than 500 mutation sites have been documented, including missense, nonsense, insertions or deletions, frameshift, and splice site mutations [10]. Notably, most CSF1R-RD-associated mutations cluster within the tyrosine kinase domain (TKD) of CSF1R. Additionally, recent studies have identified intronic mutations within the CSF1R gene [11, 12]. Our recent identification of a novel mutation locus, T567M, situated outside the TKD region [13], presents a unique opportunity to investigate pathogenic mechanisms distinct from those of classical TKD mutants. Unlike the broad kinase impairment often caused by TKD mutations, the extra-TKD location of T567M suggests that it may exert its effects through alternative mechanisms, such as the selective disruption of specific autophosphorylation sites (e.g., Y546), a hypothesis that remains entirely unexplored. Therefore, unraveling the molecular mechanism of this novel mutation is critical for a comprehensive understanding of CSF1R-RD.

CSF1R is expressed in both microglia and neurons [14, 15]. Mutations in CSF1R lead to primary microgliopathy and impaired neurogenesis [16–18]. The ligands CSF1 and interleukin-34 engage CSF1R, triggering autophosphorylation. However, CSF1R mutations disrupt autophosphorylation at critical sites such as Tyr546, Tyr708, and Tyr723, which are essential for downstream signaling initiation. Autophosphorylation of CSF1R significantly influences microglial homeostasis, neuronal survival, and neurogenesis [19, 20]. CSF1R-RD manifests as cognitive decline and movement disorders [21]. The age of onset exhibits wide variability, ranging from 18 to 78 years [3, 4]. Haploinsufficiency of CSF1R significantly impairs synaptic plasticity, leading to spatial and cognitive memory deficits [22]. Notably, specific reduction of CSF1R expression in microglia is also implicated in CSF1R-RD pathogenesis [17]. In a mouse model (CSF1R^+/−^), treatment with a CSF1R inhibitor to eliminate microglia attenuated pathological phenotypes and restored behavioral deficits [23]. These findings underscore the pivotal role of microglial dysfunction in CSF1R-RD and highlight microglial replacement therapy as an emerging research avenue. Loss-of-function of CSF1R has been extensively explored using the mouse, zebrafish, or rat models [24]. Nevertheless, a comprehensive functional characterization of the impact of CSF1R mutations on neurodegeneration in CSF1R-RD remains unexplored, particularly within the context of human induced pluripotent stem cell (iPSC)-derived microglia, neuronal cells, and cerebral organoids (COs).

In this study, we utilized CSF1R-RD patient iPSCs-derived microglia (iMGL), neurons, and COs to unravel how the CSF1R-T567M mutation contributes to CSF1R-RD. Specifically, we reprogrammed peripheral blood mononuclear cells (PBMCs) from CSF1R-RD patients harboring the CSF1R-T567M mutation into human iPSCs. Employing bulk RNA-sequencing, phagocytosis assays, migration assays, and cytokine profiling, we delineated the molecular basis of primary microgliopathy using iPSC-derived microglia. Furthermore, we evaluated neural progenitor cell (NPC) proliferation and assessed neuronal maturation, revealing neurogenesis dysfunction. Additionally, we investigated whether the CSF1R-MT correlated with the downregulation of synaptic proteins and characterized electrophysiological properties of iPSC-derived COs using whole-cell patch-clamp recordings. Our findings underscore the impact of the CSF1R-MT on microglial homeostasis, neurogenesis, and synaptic function.

## Results

### CSF1R-MT induces haploinsufficiency of CSF1R and reduces CSF1R autophosphorylation at Tyrosine 546

**Fig. 1:**
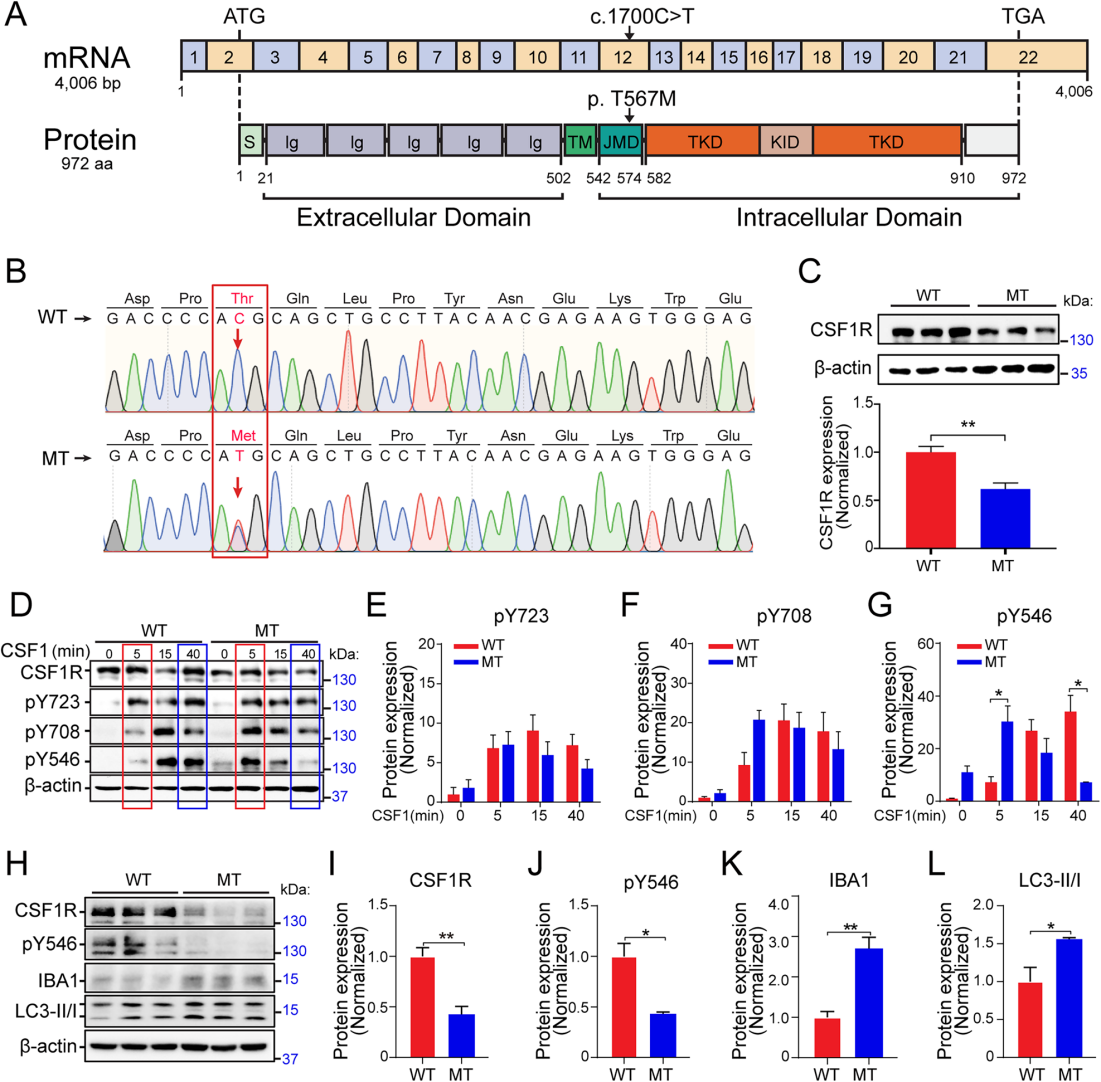
CSF1R-MT reduces CSF1R autophosphorylation at Y546 and activates autophagy in vitro. **A** CSF1R mRNA (upper panel) and protein domain structure (lower panel). The boxes in the upper panel (mRNA) are exons, and the arrow indicates the CSF1R-T567M mutation. The arrow in the lower panel (protein) indicates the position of the T567M mutation in the CSF1R protein. S signal peptide, Ig immunoglobulin-like domain, TM transmembrane domain, JMD juxta-membrane domain, TKD tyrosine kinase domain, KID kinase insert domain. **B** Sanger sequencing electropherograms of control and CSF1R-MT cDNA derived from human blood. The red arrows indicate the point mutation site at T567M in the CSF1R-RD patient. **C** SH-SY5Y cells stably expressing CSF1R-WT (WT) and CSF1R-MT (MT) were collected for western blotting. **D**–**G** SH-SY5Y cells stably expressing CSF1R-WT and CSF1R-MT were treated with CSF1 for 0, 5, 15, and 40 min before western blot analysis of pY723-CSF1R, pY708-CSF1R, and pY546-CSF1R in (**D**). Statistical analysis for pY723 (**E**), pY708 (**F**), and pY546 (**G**) from (**D**), *N* = 3. **H**–**L** BV2 cells stably expressing CSF1R-WT and CSF1R-MT were then used for western blot analysis (**H**). Statistical analysis of CSF1R (**I**), pY546-CSF1R/CSF1R (**J**), IBA1 (**K**), and LC3-II/I (**L**) from (**H**), *N* = 3. Two-tailed Student’s *t*-test was used to compare the differences between the two groups for (**C**) and (**I**–**L**). Two-way ANOVA with Tukey’s *post hoc* test was used to compare different groups for (**E**–**G**). Data are presented as mean ± SD. The statistical significance levels were set at *p < 0.05 and **p < 0.01.

Our previous work identified a pathogenic c.1700C>T (p.T567M) variant in the CSF1R gene through whole-genome sequencing analysis of CSF1R-RD cases, which was predicted in silico to be a pathogenic p.T567M mutation [25]. Until now, most genetic variants of CSF1R occur within or affect the TKD, and no pathogenic point mutation has been reported to affect regions other than the TKD [26]. Thr567 is in the juxta-membrane domain (JMD), which is just preceding the TKD (Fig. 1A). To confirm the genotype of the c.1700C>T variant of CSF1R, we first examined the CSF1R mRNA from the patient-derived blood samples. Sanger sequencing results revealed two peaks at the c.1700 locus, representing the heterozygous mutation in our CSF1R-RD case samples. One peak represents the wild-type (WT) threonine (ACG) (named CSF1R-WT or WT) and the other is the C>T point mutation, encoding the threonine-to-methionine (ATG) mutation (named CSF1R-MT, or MT) (Fig. 1B). To investigate the effect of T567M mutation on CSF1R protein expression, we generated CSF1R-WT and CSF1R-MT stably expressing neuroblastoma SH-SY5Y cells and found that the CSF1R-MT significantly reduced CSF1R protein expression (reduced 38.4%, Fig. 1C and Supplementary Fig. S1). These results suggest that the heterozygous T567M mutation in the CSF1R-RD patients causes haploinsufficiency of CSF1R, which is consistent with previous reports [27, 28].

CSF1R acts as a cell surface receptor for the CSF1 and IL-34 cytokines, which are important for cell survival and differentiation in the central nervous system [29]. The binding of CSF1 to CSF1R stimulates receptor homodimer formation and autophosphorylation of tyrosine residues in the cytoplasmic domain [30, 31]. To examine the impact of CSF1R-MT on CSF1R, we first examined the autophosphorylation of CSF1R in SH-SY5Y. After stimulation with the cytokine CSF1 for 5, 15, or 40 min, the autophosphorylation of CSF1R at Tyr723 (Y723) and Tyr708 (Y708) was not affected by the CSF1R-MT (Fig. 1D–F and Supplementary Fig. S1). Notably, the autophosphorylation of CSF1R-MT at Tyr546 (Y546) was significantly increased after 5 min treatment of CSF1 and decreased after 40 min treatment of CSF1 when compared to CSF1R-WT (Fig. 1D, G). These data suggest that the T567M mutation outside of the TKD alters CSF1R autophosphorylation at Y546.

Given that CSF1R is highly expressed in microglial cells, next, we sought to investigate the effect of CSF1R-MT in microglia. In the CSF1R-WT and CSF1R-MT stable BV2 microglial cells, which maintain a constitutively active baseline state through endogenous expression of CSF1R, CSF1R-MT significantly reduced CSF1R protein level by 57.4% and autophosphorylation of CSF1R at Y546 by 56.3% (Fig. 1H–J and Supplementary Fig. S1). The observed cell-type-specific difference in pY546 dynamics—transient increase in SH-SY5Y under acute ligand stimulation versus sustained reduction in BV2 cells under baseline conditions—likely stems from their distinct basal signaling status and experimental paradigms. We hypothesize that the T567M mutation may impair receptor internalization/degradation upon activation [32]. In SH-SY5Y cells with CSF1 stimulation, the mutant receptor exhibits a transient pY546 increase due to prolonged membrane retention before its eventual decline, whereas in BV2 cells under endogenous ligand conditions, the mutation manifests primarily as a constitutive reduction in receptor stability and basal phosphorylation. This divergence underscores the context-dependent nature of CSF1R regulation through both ligand-induced activation and intrinsic receptor stability mechanisms.

Additionally, we also detected a significant activation of microglia with IBA1 (increased 179.3%, Fig. 1H, K) and an increase in autophagy activity by the expression of autophagy-related protein LC3-II/I in CSF1R-MT compared to CSF1R-WT (increased 58.5%, Fig. 1H, L). Together, CSF1R-T567M induced haploinsufficiency of CSF1R, reduced CSF1R autophosphorylation at Tyr546, and activated autophagy in microglia.

### CSF1R-MT triggers neuroinflammation and abnormal phagocytosis and impairs migration in iMGL

**Fig. 2:**
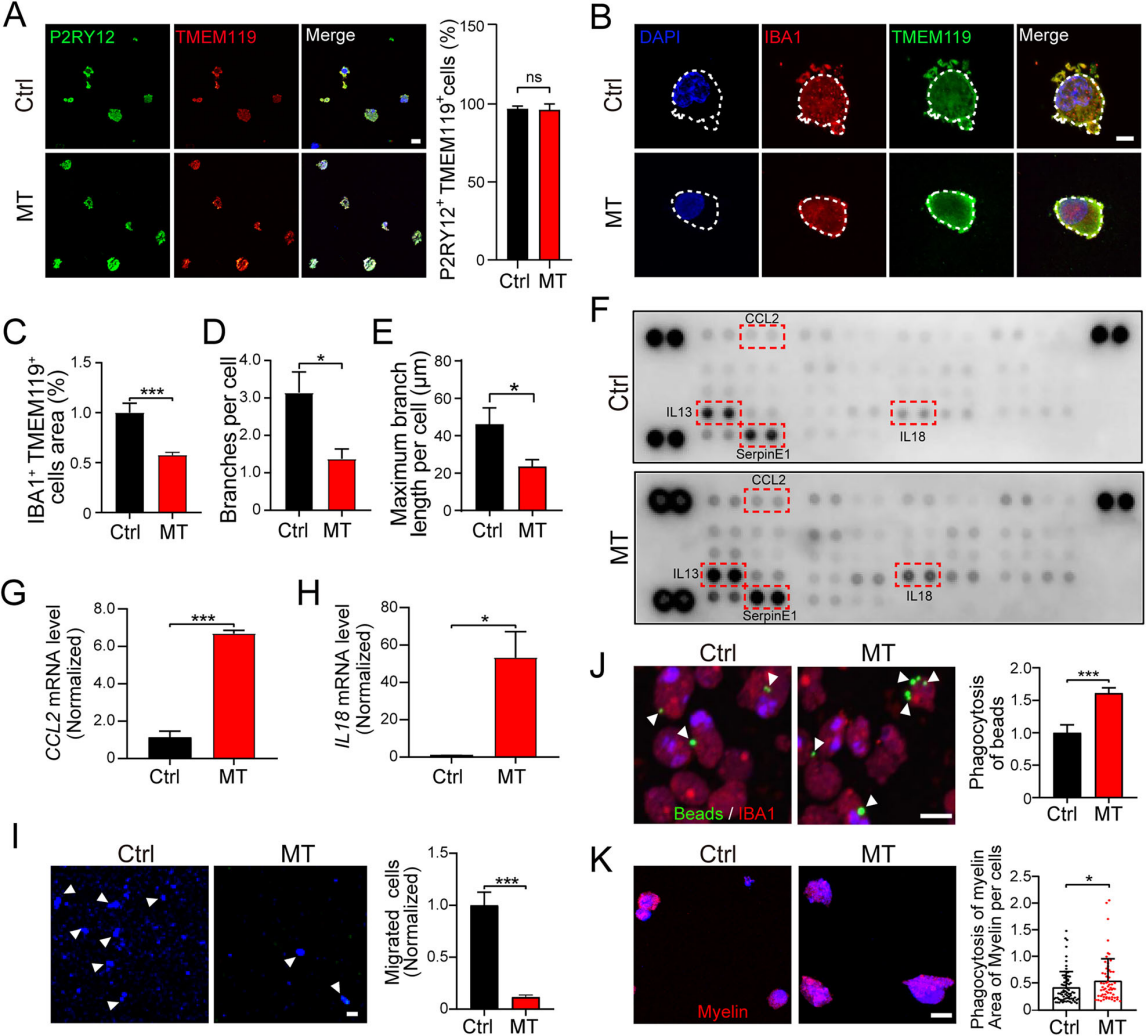
CSF1R-MT triggers neuroinflammation and phagocytosis in patient iMGL. CSF1R-T567M mutant (MT) and corrected (Ctrl) iPSCs derived from patients were used to differentiate into microglia (iMGL). **A** Representative images of P2RY12^+^ and TMEM119^+^ iMGL. Scale bar = 10 µm. **B** Comparison of area, branch number, and maximum branch length between Ctrl and MT iMGL with IBA1^+^ TMEM119^+^ staining. Cells from 50 fields of view were counted for each group. Scale bar = 5 µm. Statistical analysis of IBA1^+^ iMGL cell area (**C**), branches per cell (**D**), and maximum branch length (**E**) in (**B**). **F** Cytokine profiling assay after 24 h of stimulation with 100 ng/mL LPS, showing significant upregulation of candidate cytokines. RT-qPCR of *CCL2* (**G**) and *IL-18* (**H**) transcription in Ctrl and MT iMGL. *N* = 3. **I** Migration assay of Ctrl and MT iMGL after stimulation with ATP. White arrowheads indicate some of the migrated iMGL. Cells from 15 fields of view were counted for each group. Scale bar = 5 µm. **J** Effect of the CSF1R-MT on the phagocytic ability of green fluorescent latex beads (white arrowheads). The iMGL were stained with IBA1 (red signals). *N* = 8. Scale bar = 10 µm. **K** Effect of the CSF1R-MT on the phagocytic ability of the pHrodo-labeled myelin (red signals) in iMGL. Number of cells quantified: Ctrl = 76, MT = 61. Scale bar = 10 µm. Two-tailed Student’s *t*-test was used to compare the differences between the two groups. Data are presented as mean ± SD. The statistical significance levels were set at *p < 0.05 and ***p < 0.001.

Microglia are brain-resident macrophages with phagocytic and immune surveillance functions. CSF1R is mainly expressed in microglia and regulates their proliferation and development [10, 33], suggesting that microglial dysfunction plays an important role in CSF1R-RD pathogenesis [26]. To better understand the T567M mutation’s effects on microglial function, PBMCs were isolated from clinical blood samples and reprogrammed into iPSCs. Isogenic control lines (Ctrl) were then generated from CSF1R-MT iPSCs (MT) using CRISPR/Cas9-mediated genome editing. We first induced iPSCs into hematopoietic progenitor cells (HPC), then gradually differentiated and matured them into microglia using the published protocol [34]. To characterize patient iMGL, day 46 (D46) iMGL were collected and immunostained with TMEM119 and IBA1, which are common microglia markers [35]. Immunostaining revealed mature iMGL to be more than 96% (Ctrl: 97.0%; MT: 96.4%) double positive for P2RY12 and TMEM119 (Fig. 2A), the positive rates are similar to those reported in the other research [36]. These results indicate the successful differentiation of iPSCs into iMGL. To further characterize the pathological state of iMGL, we examined the morphology of iMGL. We observed that the area of MT iMGL was significantly smaller compared to the Ctrl iMGL by IBA1 staining (Fig. 2B, C). We also found the surface area of MT iMGL was less elongated, smaller, and more compact, compared to control iMGL. In addition, we found that Ctrl iMGL had more prominent fine filopodia with more ramified surface than MT iMGL (Fig. 2B). There were fewer branches per cell and shorter maximum branches per cell in MT compared to Ctrl iMGL (decreased 56.3 and 49.2%, respectively, Fig. 2D, E). These morphological changes indicate a hyperactive pathological state of MT iMGL induced by the T567M mutation.

Microglial state plays an important role in neuroinflammatory response in the central nervous system [37]. To further investigate the pathophysiological function of iMGL, we first examined the immune cytokine activity of iMGL with LPS stimulation. Results showed that MT iMGL secreted detectable levels of several cytokines and chemokines, including CCL2, IL-13, IL-18, and serpinE1 (Fig. 2F). Among these, CCL2 and IL-18 can impair the microenvironment of the brain by initiating an inflammatory response [38, 39]. Quantitative PCR data confirmed that the expression levels of *CCL2* and *IL-18* were significantly increased in MT compared to Ctrl iMGL after stimulation (increased 5.0-fold and 51.7-fold, respectively, Fig. 2G, H), indicative of a hyperreactive state of MT iMGL. Migration is another important neuroinflammatory property in the immune system. Once brain injury occurs, microglia migrate to the lesion site and take part in the immunoreaction [40]. Thus, we next examined the migration of patient iMGL in a transwell assay. Results showed that there were significantly fewer migrated iMGL in MT compared to Ctrl (decreased 88.2%, Fig. 2I). This finding is consistent with established knowledge, as impaired migration has been identified as a hallmark functional deficit in stem cell-derived microglia models of CSF1R-related disorders [36]. We next investigated phagocytosis, a process crucial for synaptic homeostasis. Proper phagocytosis of microglia is conducive to the regulation of synaptic plasticity, while excessive phagocytosis could impair synaptic function, which is closely related to CSF1R-RD [10, 35, 41]. To assess the phagocytic capacity of patient-derived iMGL, we co-cultured cells with fluorescent latex beads. MT iMGL exhibited a significantly higher phagocytic activity compared with Ctrl iMGL, showing a 61.1% increase (Fig. 2J). Consistently, MT iMGL demonstrated enhanced uptake of pHrodo-labeled myelin isolated from mouse brains following 6 h of exposure, with a 30.18% increase relative to Ctrl iMGL (*p* = 0.0443; Fig. 2K). A similar trend was observed in the human microglial cell line HMC3. MT HMC3 cells displayed significantly increased uptake of pHrodo-labeled myelin compared with Ctrl HMC3 cells after 6 h of exposure (138.0% increase, *p* = 0.0132; Supplementary Fig. S2). These results indicate enhanced phagocytic activity in MT microglial models, which may be closely related to the synaptic dysfunction observed in CSF1R-RD [42]. Taken together, our data suggest that CSF1R-MT triggers neuroinflammation, aberrant phagocytosis, and impairs migration in iMGL, consistent with the clinical features of CSF1R-RD [15, 26].

### Transcriptomic analysis reveals neuroinflammation in MT iMGL

**Fig. 3:**
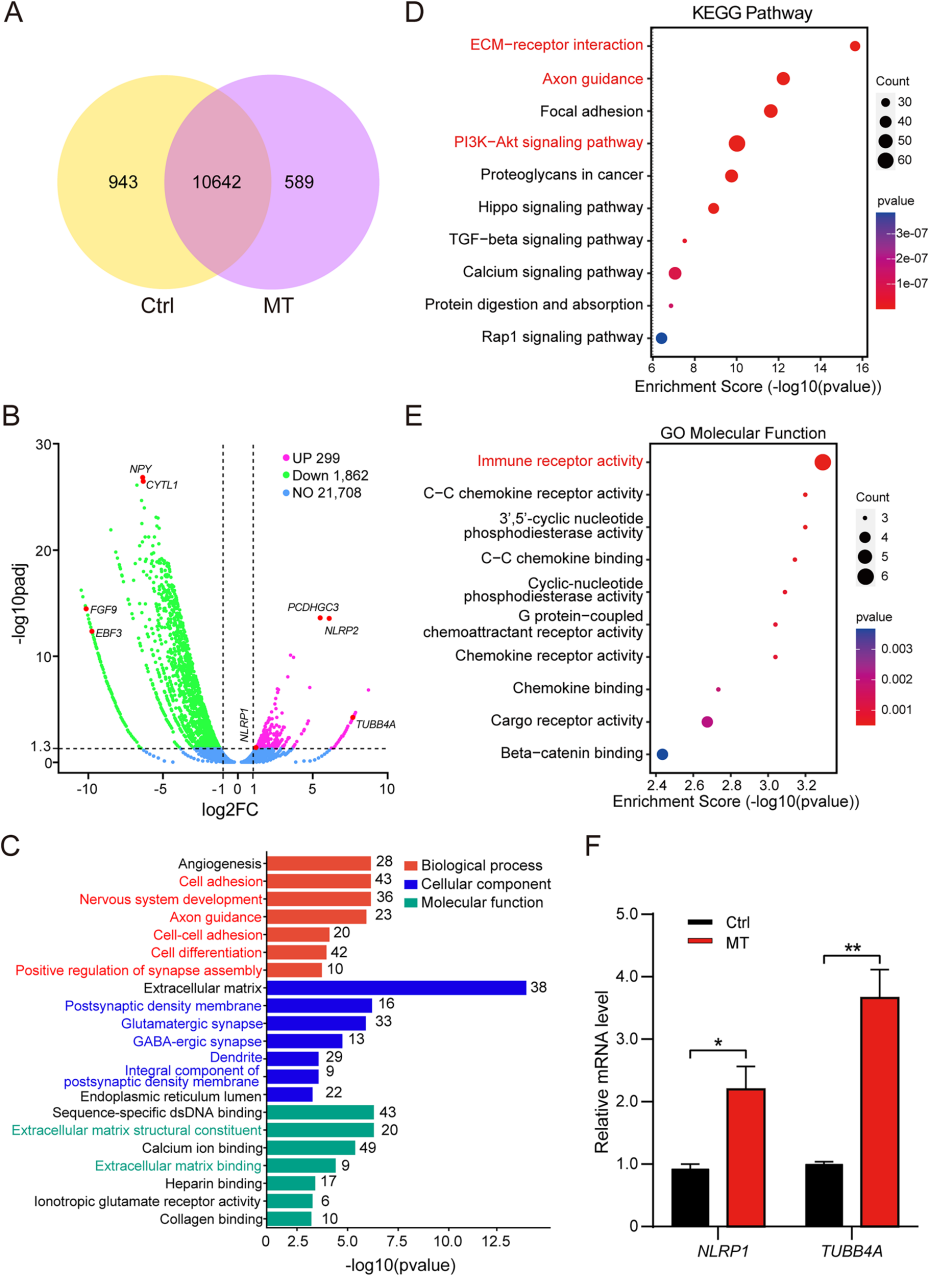
Transcriptomic analysis reveals neuroinflammation in CSF1R-MT iMGL. Three batches of D30 iMGL were used to conduct bulk RNA-sequencing. **A** Venn diagram showing co-expressed and independently expressed genes in Ctrl and MT iMGL. **B** Volcano plot of differentially expressed genes between Ctrl and MT iMGL. **C** Gene Ontology analysis of top-downregulated genes. Some biological processes, cellular components, and molecular functions are downregulated in the MT iMGL. **D** KEGG pathway analysis of downregulated genes. ECM-receptor interaction, axon guidance, and PI3K-Akt signaling pathway are downregulated in CSF1R-MT iMGL. **E** Gene Ontology analysis of upregulated genes. Immune receptor activity is upregulated in the MT iMGL. **F** qPCR analysis of *NLRP1* and *TUBB4A* mRNA expression. Two-tailed Student’s *t*-test was used to compare the differences between the two groups, *N* = 3. Data are presented as mean ± SD. The statistical significance levels were set at *p < 0.05 and **p < 0.01.

To better understand the molecular profile of CSF1R-MT iMGL in CSF1R-RD, we conducted bulk RNA-sequencing to investigate the transcriptomic profile in iMGL. We found that 10,642 genes were commonly expressed in both Ctrl and MT iMGL (Fig. 3A). Among those commonly expressed genes, 1862 genes were downregulated and 299 genes were upregulated in MT iMGL. Notably, several key downregulated genes included *NPY*, *CYTL1*, *FGF9*, and *EBF3*, whereas upregulated genes comprised *PCDHGC3*, *NLRP2*, *TUBB4A*, and *NLRP1* (Fig. 3B). Gene ontology (GO) analysis of these downregulated genes revealed several downregulated biological processes in MT iMGL, including cell adhesion, nervous system development, axon guidance, cell-cell adhesion, cell differentiation, and positive regulation of synapse assembly (Fig. 3C). Decreased cell adhesion is related to impaired migration, and abnormalities in axon guidance and regulation of synapse assembly may be important for cognitive impairment in CSF1R-RD [43]. Defects in cell differentiation might lead to a reduction in the size and branches of iMGL, which aligns with our observations (Fig. 2B–E). GO cellular component analysis identified downregulated genes enriched in postsynaptic density membrane, glutamatergic synapse, GABAergic synapse, dendrite, and integral component of postsynaptic density membrane (Fig. 3C). These cellular components are highly relevant to microglial synaptic pruning, indicative of synaptic dysregulation in MT iMGL. GO molecular function analysis also detected dysfunction in the extracellular matrix (ECM) structural component and ECM binding, which is associated with migration deficiency of MT iMGL (Fig. 3C). In addition, several KEGG pathways were also found to be downregulated in CSF1R-MT iMGL, including axon guidance, ECM-receptor interaction, and PI3K-Akt signaling (Fig. 3D).

When analyzing the upregulated genes, we found that they were mainly enriched in immune receptor activity in MT iMGL, with noteworthy enrichment in immune receptor activity (Fig. 3E), which supports the hyperreactive state of the MT iMGL after stimulation (Fig. 2F). Among the differentially expressed gene list, *NLRP1* is one of the most important inflammasomes involved in a variety of neurodegenerative diseases [44, 45]. *TUBB4A* is closely related to leukodystrophy [46, 47]. Our qPCR data confirmed that *NLRP1* and *TUBB4A* were significantly increased in the MT iMGL (increased 1.37-fold and 2.67-fold for NLRP1 and TUBB4A, respectively, Fig. 3F). Altogether, the transcriptomic profile analysis reveals that several important biological processes, cellular components, molecular functions, and pathways are involved in CSF1R-MT-induced microglial dysfunction, including defects in synapse function and neuroinflammation.

### CSF1R-MT promotes neural stem cell proliferation but inhibits neuronal maturation

**Fig. 4:**
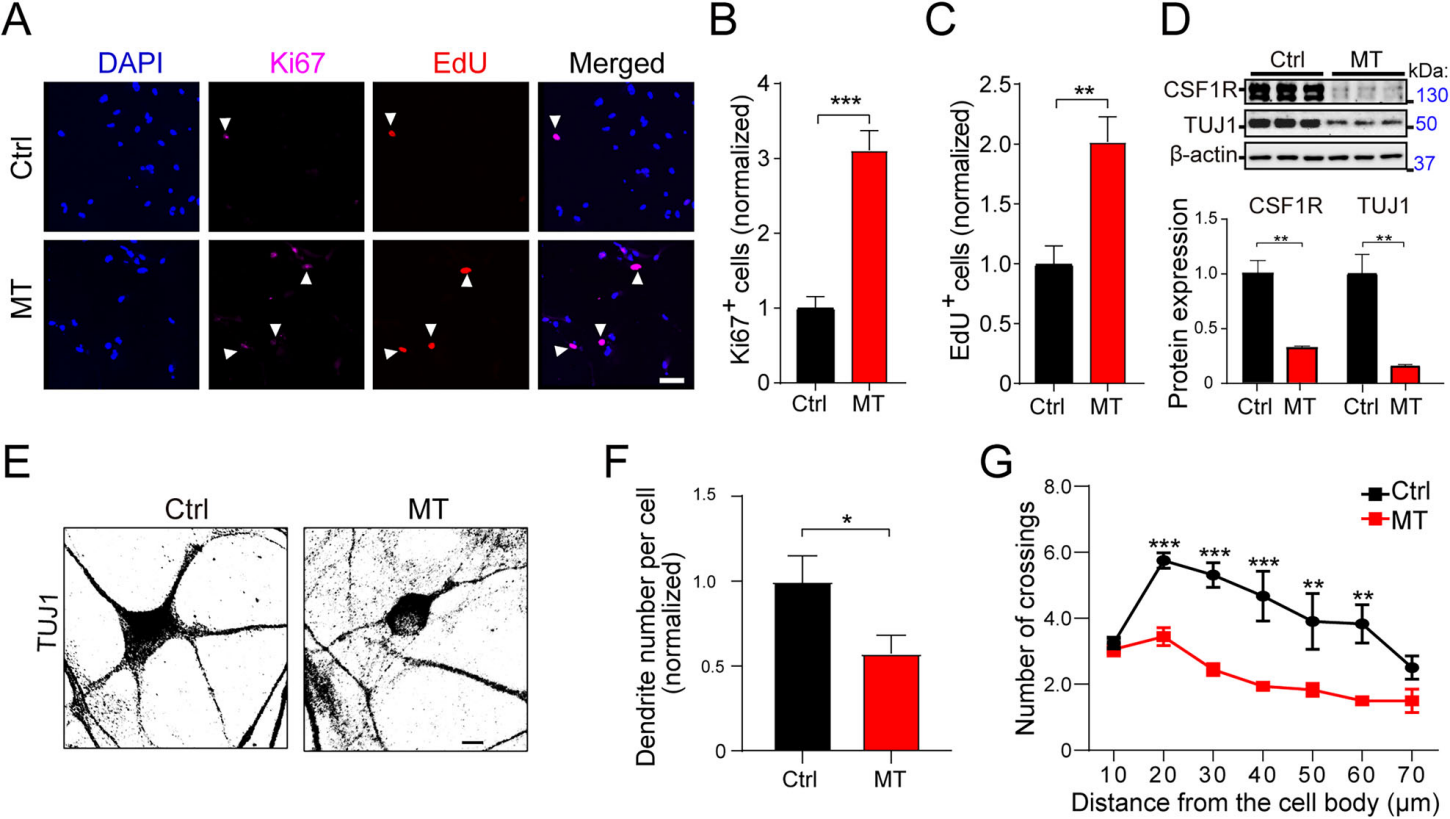
CSF1R-MT promotes neural proliferation but inhibits neuronal differentiation and maturation. NPCs and neurons differentiated from the Ctrl and MT iPSCs were used to study the effect of CSF1R-MT on neuronal functions. **A**–**C** NPCs were stained with Ki67 and EdU. **A** White arrowheads indicate Ki67/EdU double-positive NPCs. Scale bar = 25 μm. Statistical analysis of Ki67-positive NPCs (**B**) and EdU-positive NPCs (**C**) in (**A**). Cells from 18 fields of view were counted for each group. **D** Western blot analysis of CSF1R and TUJ1 in Ctrl and MT iPSC-derived neurons. *N* = 3. **E** Representative images of Ctrl and MT iPSC-derived neurons (D19), stained by TUJ1. Sholl analysis of dendrite number per cell (**F**) and number of crossings (**G**) in (**E**). *N* = 7. Scale bar = 5 μm. Two-tailed Student’s *t*-test was used to compare the two groups. Data are presented as mean ± SD. The statistical significance levels were set at *p < 0.05, **p < 0.01, and ***p < 0.001.

CSF1R not only regulates microglia activity but also plays an important role in the physiological function of neurons [15]. Our RNA-seq data indicated synaptic dysfunction in MT iMGL; thus, we next examined the role of CSF1R-MT in neural proliferation and differentiation. iPSC-derived NPCs were immunostained with Ki67 and EdU, two proliferation markers [48–51]. We found that the T567M mutation increased the number of both Ki67^+^ and EdU^+^ cells (increased by 210% and 102%, respectively, Fig. 4A–C), indicating that CSF1R-MT promotes NPC proliferation. We further differentiated NPCs into neurons and found downregulation of TUJ1 (βIII-tubulin, a well-established neuronal marker) in MT compared to Ctrl neurons (decreased 84.1%, Fig. 4D and Supplementary Fig. S3). This defect was associated with a significant reduction in CSF1R expression (decreased 67.7%, Fig. 4D), supporting the notion that CSF1R haploinsufficiency underpins aberrant neuronal development. Furthermore, the observed reduction in TUJ1^+^ neurons was not attributable to increased apoptosis, as evidenced by comparable levels of Cleaved Caspase-3 (CC3) between MT and Ctrl neurons (Supplementary Fig. S4). In addition, we analyzed the changes in dendritic complexity of neurons by Sholl analysis to assess neural maturation [49]. We found that dendrite number (43.1% decreased, Fig. 4E, F) and the number of crossings at distances of 20–60 µm from the cell body were significantly decreased in the MT neurons compared to the Ctrl neurons (Fig. 4E, G). Our results indicate that CSF1R-MT promotes NPC proliferation but inhibits neural differentiation and delays neural maturation.

### CSF1R-MT delays neurodevelopment of COs and impairs synaptic function

**Fig. 5:**
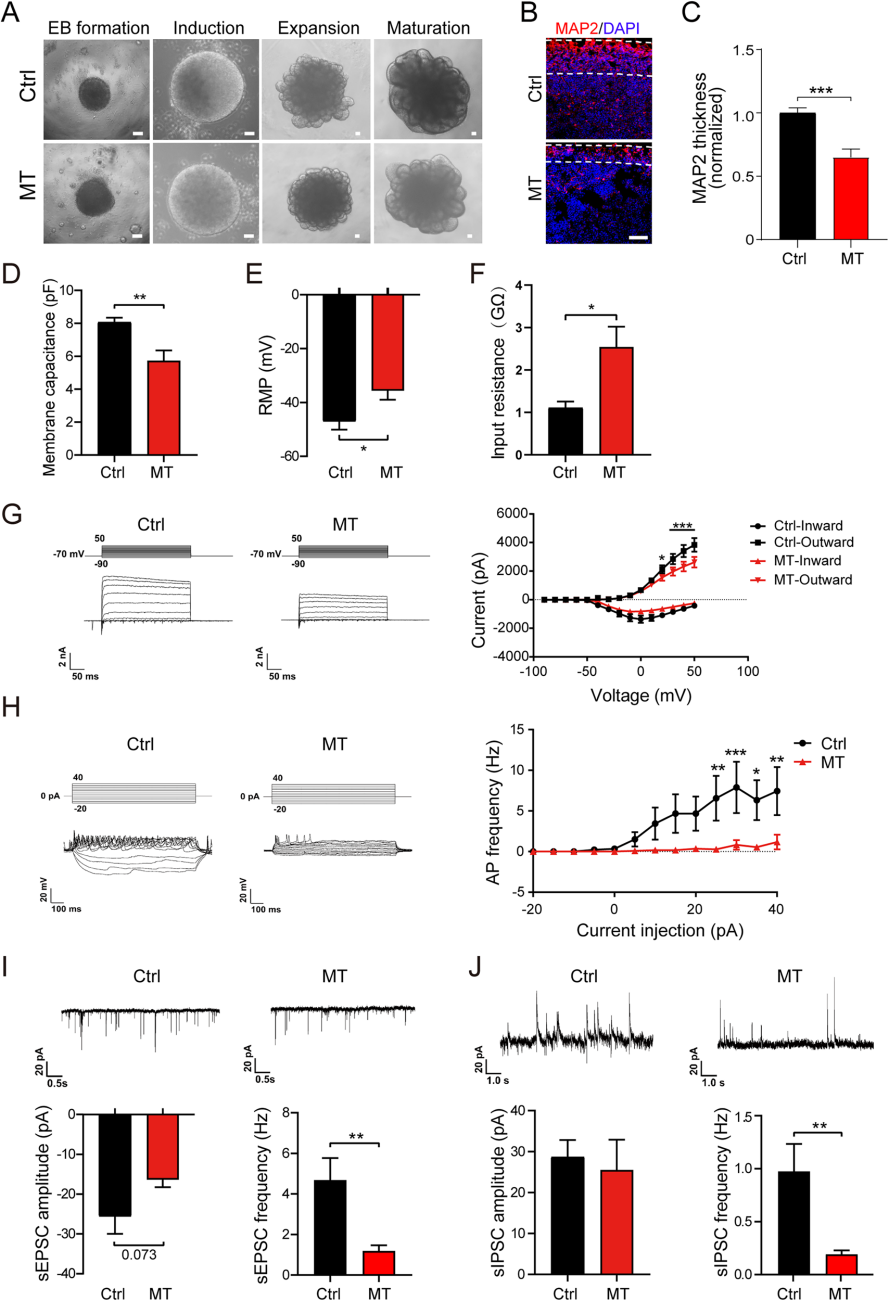
CSF1R-MT delays neurodevelopment and impairs synaptic function in COs. **A** Representative images of each stage of cerebral organoid (CO) generation. **B**, **C** Immunofluorescence staining of MAP2 at day 60 in MT and Ctrl COs. *N* = 10. **D**–**J** D130 COs were used for whole-cell patch-clamp recordings. *N* = 11. The membrane capacitance (**D**), resting membrane potential (RMP) (**E**), and input resistance (**F**) were recorded. **G** Representative traces and quantification of voltage-gated currents in MT and Ctrl COs. **H** Analysis of neuronal firing ability in MT and Ctrl COs. **I** Representative traces and quantification of spontaneous excitatory postsynaptic current (sEPSC) in MT and Ctrl COs. **J** Representative traces and quantification of spontaneous inhibitory postsynaptic current (sIPSC) in CSF1R-MT and Ctrl COs. Two-tailed Student’s *t*-test was used to compare the differences between the two groups (**C**, **D**–**F**, **I**, **J**). Two-way ANOVA with Tukey’s *post hoc* test was used to compare the two groups (**G**, **H**). Data are presented as mean ± SD. The statistical significance levels were set at *p < 0.05, **p < 0.01, and ***p < 0.001.

Given that the cerebral cortex is the most evolutionarily extended region of the human brain [52], we next sought to use COs to study the effect of CSF1R-MT on neurodevelopment. We followed the established protocol to generate COs [52] (Fig. 5A). On day 60 (D60), we found that the expression of MAP2 (microtubule-associated protein 2, a mature neuronal marker) was significantly decreased in MT COs (decreased 35.0%, Fig. 5B, C), which indicates that CSF1R-MT impairs neurogenesis of COs. This is consistent with the 2D neurogenesis study (Fig. 4). To investigate whether CSF1R-MT induces neuron functional changes in COs, we performed whole-cell patch-clamp recordings of COs on day 130 (D130). The membrane capacitance indicates the size of recorded neurons. We found that the size of MT neurons in COs was significantly decreased compared to the corresponding control ones (decreased by 29.1%, Fig. 5D). The significant reduction in membrane capacitance and MAP2 expression suggests structural simplification in mutant neurons, potentially reflecting impaired dendritic arborization or reduced synaptic density. The resting membrane potential (RMP) and input resistance of MT COs were significantly increased (increased 24.2 and 132%, respectively, Fig. 5E, F), which indicates that neuronal maturity and the expression of ion channel proteins were potentially decreased compared to the Ctrl. We next detected the voltage-gated current; results showed that the outward potassium current of MT COs was significantly decreased at 20–50 mV while there was no significant difference in the inward sodium current (Fig. 5G). The firing ability and synaptic transmission function are the most important physiological functions of neurons. We further explored whether CSF1R-MT affects neuronal firing ability and detected the action potential (AP) frequency, which was subjected to increasingly positive current injection from −20 to 40 pA with 5 pA increments. The results showed that the AP frequency of the neurons in MT COs was significantly decreased compared to the Ctrl ones at 25–40 pA (Fig. 5H), which may suggest multiple potential ion channel dysregulations. Meanwhile, we measured the spontaneous excitatory postsynaptic current (sEPSC) and spontaneous inhibitory postsynaptic current (sIPSC) frequency and found that there were significantly decreased sEPSC and sIPSC in MT compared to the Ctrl COs (decreased 74.6% and 80.9%, respectively, Fig. 5I, J). The results demonstrate that the CSF1R-MT impairs synaptic function, which is in alignment with previous observations in neurons and microglia (Figs. 3 and 4). Our findings provide a potential explanation for the cognitive symptoms in CSF1R-RD, in which intrinsic neuronal defects and non-cell-autonomous microglial effects collectively contribute to the disease process.

### CSF1R-MT iMGL exacerbates synaptic protein deficits in co-cultured MT COs

**Fig. 6:**
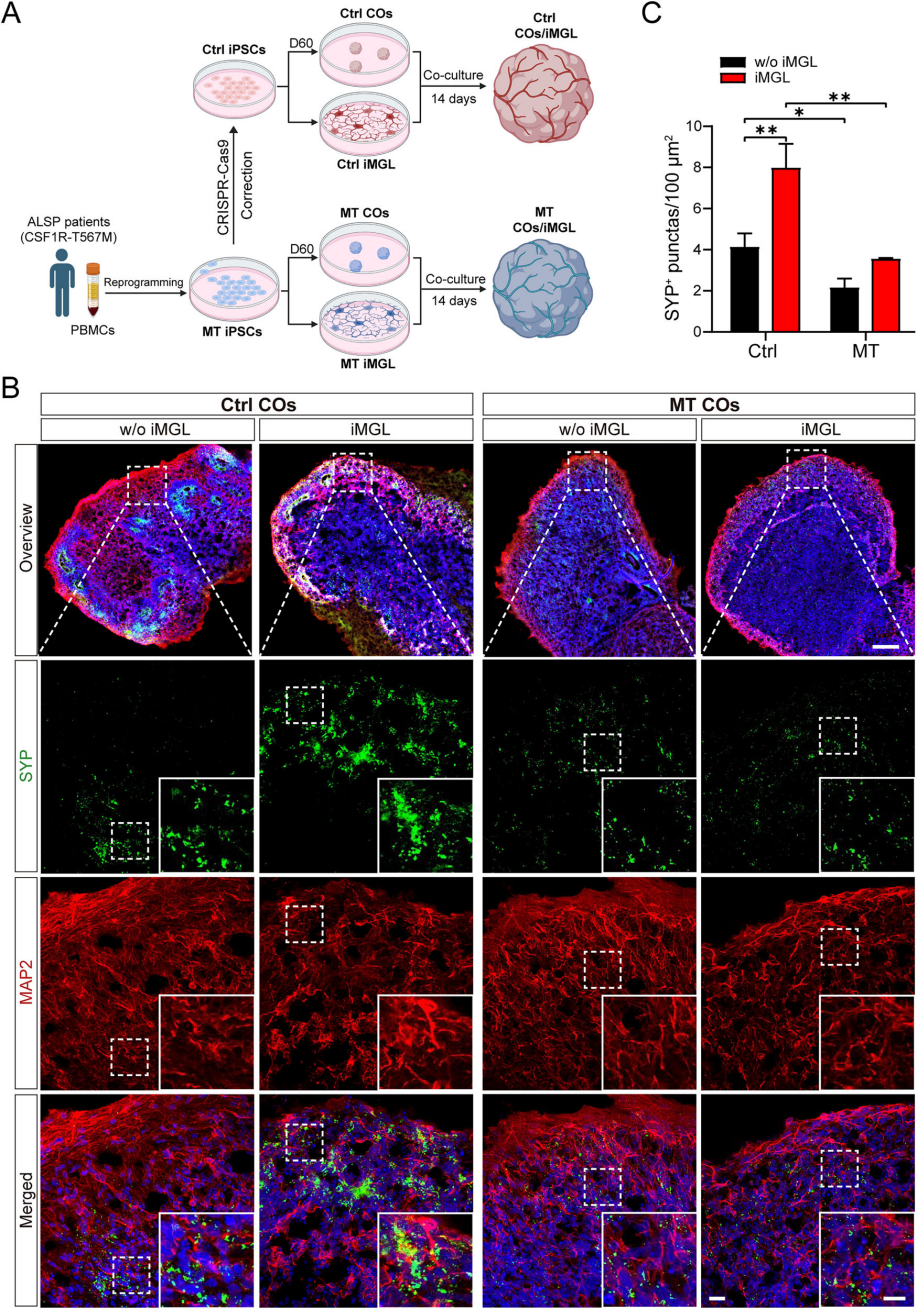

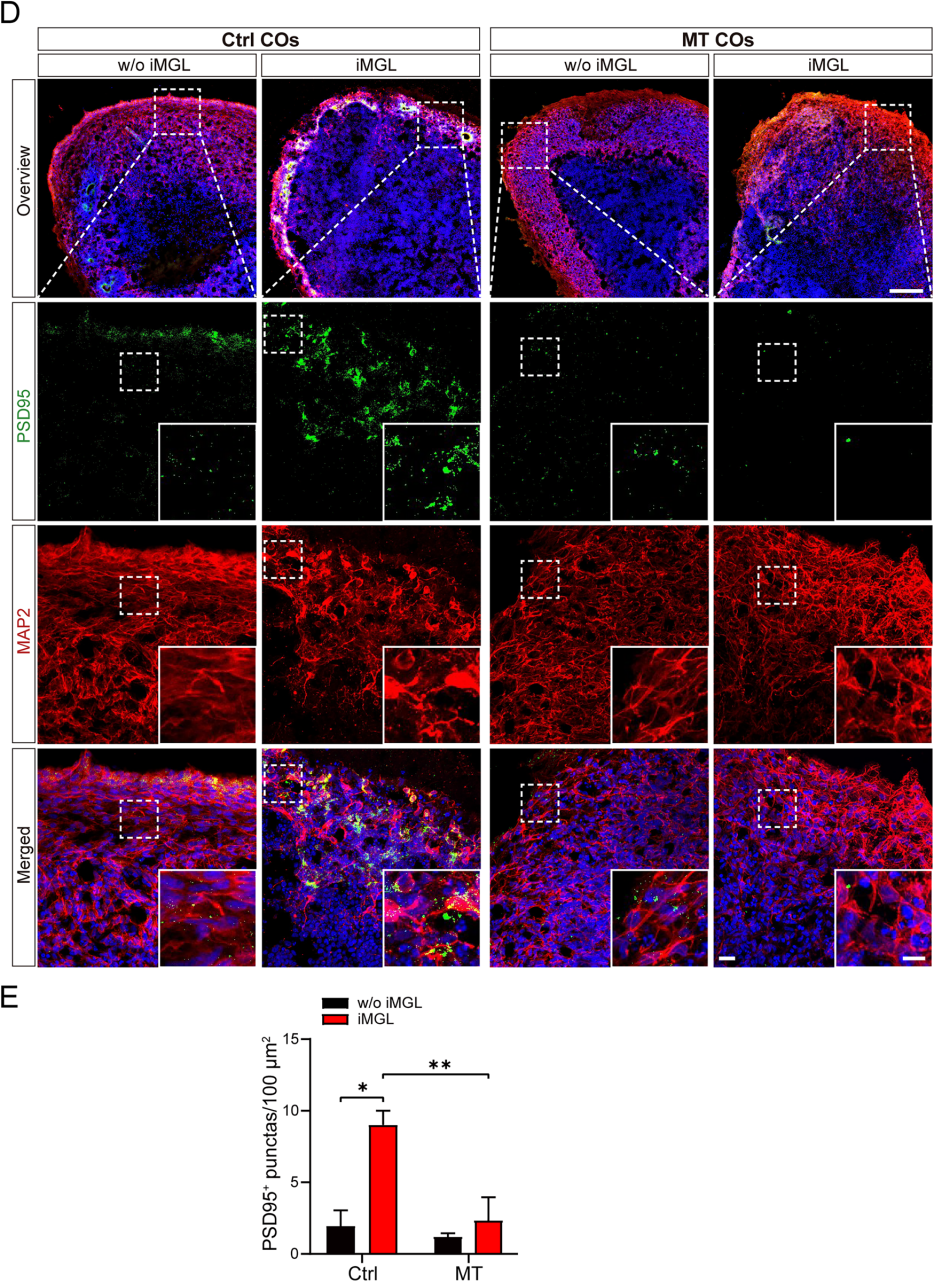
CSF1R-MT iMGL exacerbates synaptic protein deficits in co-cultured MT COs. **A** Schematic of the co-culture procedure of patient-derived iMGL and COs. **B** COs (D60) co-cultured with or without (w/o) iMGL for 14 days were used for cryosectioning, followed by immunostaining with synaptophysin and MAP2. Scale bar = 200 µm in the overview image; 20 µm and 10 µm in the large and zoomed-in images (merged channels). **C** Statistical analysis of synaptophysin^+^ puncta shown in (**B**), *N* = 3. **D** COs co-cultured with iMGL for 14 days or without (w/o) iMGL were used for cryosectioning, followed by immunostaining with PSD95 and MAP2. Scale bar = 200 µm in the overview image; 20 µm and 10 µm in the large and zoomed-in images (merged channels). **E** Statistical analysis of PSD95 shown in (**D**), *N* = 3. Two-way ANOVA with Tukey’s *post hoc* test was used to compare groups. Data are presented as mean ± SD. The statistical significance levels were set at *p < 0.05 and **p < 0.01.

CSF1R is expressed in microglia and neurons, both of which play an important role in CSF1R-RD pathogenesis. To investigate iMGL function within a specific genetic context, we established isogenic co-culture systems, with Ctrl iMGL co-cultured with Ctrl organoids, and MT iMGL co-cultured with MT organoids for 14 days (Fig. 6A). We then assessed the neuronal synaptic integrity by immunostaining the presynaptic marker synaptophysin (SYP) and the postsynaptic marker postsynaptic density protein 95 (PSD95) [53]. Results showed that MT organoids exhibited a baseline reduction in SYP expression, consistent with intrinsic neurodevelopmental delay and synaptic function impairment observed in MT COs (Fig. 5). Co-culture experiments further revealed that, in the WT context, Ctrl iMGL significantly upregulated SYP levels compared to Ctrl organoids without iMGL co-culture (increased by 116.8%, Fig. 6B, C). In contrast, this beneficial effect of microglia was abolished in MT organoids co-cultured with MT iMGL, which displayed a marked reduction in SYP levels compared to Ctrl co-cultured organoids (reduced by 48.4%, Fig. 6B, C). Similarly, PSD95 expression was significantly enhanced in Ctrl COs with iMGL compared to Ctrl COs without iMGL (increased 3.79-fold, Fig. 6D, E), whereas PSD95 levels were markedly reduced in MT co-cultured COs compared to Ctrl co-cultured COs (reduced by 74.8%, Fig. 6D, E). The functional state of iMGL is a decisive factor regulating synaptic protein levels [54]. The functional impairment of MT iMGL is directly associated with the reduced synaptic protein levels in the co-culture system, indicating that microglial dysfunction is a core mechanism contributing to the disease-associated synaptic abnormalities. This is closely linked to CSF1R-RD-related learning and memory impairment.

## Discussion

The CSF1R gene is implicated in the pathogenesis of CSF1R-RD, and over 500 mutation sites within CSF1R have been identified [26]. Notably, a novel T567M mutation located outside the TKD of CSF1R was characterized in this study. To understand the underlying mechanisms, researchers have employed animal and histological models. However, caution is warranted due to species-specific differences that may yield unpredictable physiological outcomes [55]. Consequently, drug development based on such models may face clinical challenges. In our investigation, we leveraged patient-derived models by reprogramming patient PBMCs into iPSCs, which were subsequently corrected by CRISPR/Cas9 genome editing. We further differentiated iPSCs into neurons, iMGL, and COs. These 2D and 3D models retain critical genetic information from CSF1R-RD patients, making them valuable tools for dissecting pathological processes and potentially aiding drug screening. Notably, the 3D model, which simulates the complexity of the human brain, was employed to explore CSF1R-RD pathogenesis in this study.

Our study represents the first functional characterization of the T567M mutation in a human iPSC model, firmly establishing that CSF1R-RD pathogenesis can arise from mutations outside the TKD through mechanisms distinct from those of classical TKD mutants. While traditional TKD mutations often lead to substantial loss of kinase activity [56], our data reveal that the T567M mutation results in a more selective functional deficit. Specifically, we demonstrated that T567M preferentially impairs autophosphorylation at residue Y546, while leaving phosphorylation at other critical sites relatively intact. This nuanced mechanism, contrasting with the broad kinase inactivation caused by typical TKD mutations, underscores the uniqueness of the T567M allele and expands our understanding of the spectrum of CSF1R dysfunction. Our findings highlight that the genetic and functional heterogeneity of CSF1R-RD is greater than previously appreciated, necessitating mutation-specific functional assessments for precise mechanistic insights.

The *CSF1R* gene is predominantly expressed in microglia, the resident immune cells of the central nervous system. Disruption of CSF1R function can lead to primary microgliopathy. In CSF1R-RD patients, the number of microglia is significantly reduced [57]. Biallelic mutations in CSF1R result in the elimination of microglia, leading to severe clinical manifestations that typically begin in childhood [58]. In the CSF1R^−/−^ mice, microglia are virtually absent [17]. Conversely, in CSF1R^+/−^ mice, the remaining microglia exhibit aberrant activation and produce inflammatory cytokines [23]. The T567M mutation of CSF1R leads to an approximately 50% reduction in CSF1R expression in microglia, contributing to their abnormal activation.

The CSF1 ligand binds to its receptor, CSF1R, initiating autophosphorylation of the receptor’s tyrosine kinase domain. Our findings demonstrate that the T567M mutation exerts a phosphorylation-specific and cell context-dependent effect on CSF1R, most notably by altering the autophosphorylation dynamics at Y546. The specific impact on Y546, rather than the canonical autophosphorylation sites Y708 and Y723, suggests a unique allosteric mechanism [59]. We postulate a potential structural basis for this phenomenon: threonine 567 is located in the juxta-membrane domain, a critical region known to regulate receptor kinase activity through autoinhibitory interactions [60]. The substitution of threonine with the more hydrophobic and structurally distinct methionine (T567M) could perturb the local conformation or destabilize autoinhibitory contacts. This structural perturbation may, in turn, allosterically modulate the flexibility or accessibility of the kinase domain loop where Y546 resides, thereby favoring its initial phosphorylation upon ligand binding in SH-SY5Y cells, while concurrently destabilizing the receptor protein, as observed in BV2 cells. Future studies employing molecular dynamics simulations could directly test this hypothesis by modeling atomic-level interactions within the juxta-membrane and kinase domains, comparing the structural networks and energy landscapes of the wild-type and T567M mutant receptors. Such simulations would be instrumental in revealing how a distal mutation outside the kinase domain can precisely influence the phosphorylation efficiency of a specific tyrosine residue, providing a mechanistic link between the mutation and the distinct signaling phenotypes we observed.

This autophosphorylation event activates downstream signaling pathways, including the PI3K and JNK pathways [12, 61]. Notably, mutations within the CSF1R tyrosine kinase domain, such as I662T, G765D, D778E, I794F, and M875T, have been identified and shown to inhibit CSF1R autophosphorylation [20]. Specifically, the T567M mutation in CSF1R disrupts autophosphorylation at Tyr546 and downregulates gene expression related to the PI3K-AKT pathway. CSF1R signaling orchestrates critical processes in microglia, including proliferation, migration, and differentiation, both in health and disease. However, CSF1R-MT impairs microglial differentiation and migration. Importantly, microglial dysfunction is associated with demyelination in CSF1R-RD [43]. The myelin sheath, which envelops neuronal axons, ensures efficient transmission of electrical impulses, maintaining normal physiological functions, including cognition [43]. Intriguingly, CSF1R haploinsufficiency leads to significant upregulation of phagosome-related genes in microglia, resulting in enhanced microglial phagocytosis of myelin [22]. Although our study did not directly assess myelin phagocytosis, the observed enhanced phagocytosis of fluorescent latex beads may serve as an indirect indicator of microglial activity due to the T567M mutation. The successful modeling of such specific microglial dysfunctions in vitro underscores the potential of iPSC-derived microglia as a platform for developing and screening future therapeutic strategies, such as CNS-wide delivery of disease-modifying proteins [62].

The CSF1R gene is also expressed in NPCs and plays a pivotal role in neurogenesis, the process by which new neurons are generated in the brain [63]. Notably, CSF1R mutations impact the proliferation and differentiation of NPCs. Deletion of CSF1R leads to increased NPC proliferation and apoptosis, along with reduced neuronal differentiation [64]. Mice with specific CSF1R deletion in NPCs exhibit an expansion of forebrain neural progenitors [18]. In our study, the CSF1R-MT enhances NPC proliferation but impairs neuronal differentiation. In addition, microglia could indirectly influence neurogenesis by modulating neural networks. Selective inhibition of microglial function can restore normal neuronal differentiation [65].

Synapse remodeling is a dynamic process that commences early in life and persists throughout the lifespan. It plays a pivotal role in learning and memory formation. However, aberrant synaptic function can result in cognitive impairment [66]. Cognitive impairment often represents an early clinical sign of CSF1R-RD. Our electrophysiological characterization of CSF1R-MT cerebral organoids revealed profound neuronal dysfunction, with altered intrinsic membrane properties and synaptic transmission deficits that may underlie the cognitive impairments observed in CSF1R-RD. CSF1R in neurons could directly regulate ion channel expression or synaptic scaffolding proteins (e.g., PSD95). Microglia actively participate in regulating synaptic plasticity [67]. They achieve this by modulating the ECM and influencing presynaptic markers in microglia-specific monoallelic CSF1R knockout models [23]. In CSF1R-RD mouse models (CSF1R^+/−^ mice), reduced CSF1R levels are associated with early synaptic plasticity impairment and concomitant spatial and cognitive deficits [22]. Our data demonstrate that MT iMGL is deficient in supporting synaptic protein levels in organoids. While the exact mechanism remains to be fully elucidated, several non-exclusive pathways can be hypothesized based on our results and established literature. First, given the critical role of CSF1R signaling in maintaining microglial homeostatic function, its impairment in MT iMGL likely disrupts the production and release of key neurotrophic factors (e.g., BDNF, IGF-1) [68], creating a less supportive microenvironment for synaptic maintenance. Second, aberrant microglial activity, such as dysregulated synaptic pruning mediated by the complement system, e.g., C1q, C3 [69], could lead to excessive elimination of synapses. Third, although an overt inflammatory signature was not predominant, subtle shifts in cytokine release could exert direct synaptotoxic effects.

Our bulk RNA-seq data on iMGL provide deeper, multi-layered mechanistic insights that extend beyond these canonical pathways. We discovered a significant downregulation in the “ECM-receptor interaction” pathway, coupled with a reduction in genes encoding an “integral component of the postsynaptic density membrane.” These findings together outline a novel, coherent cascade: the impaired ability of MT iMGL to synthesize and remodel the extracellular matrix renders them unable to provide a stable, synaptogenic niche for neurons. It is well established that ECM-integrin signaling is crucial for synaptic maturation and stability [70], directly activating intracellular pathways that regulate the stability of PSD95 and the assembly of the entire PSD. Therefore, the observed transcriptional suppression of neuronal postsynaptic components is likely a direct consequence of the defective ECM microenvironment created by MT iMGL.

This study, by leveraging an isogenic model, provides the first mechanistic insights into the CSF1R-T567M mutation, a primary goal of our work. The pathomechanisms we delineate here establish a foundational hypothesis for CSF1R-RD. Future validation and expansion of these findings require iPSC models derived from a broader spectrum of patients harboring diverse CSF1R mutations. Such efforts will be crucial to define the universal hallmarks of CSF1R dysfunction and to decipher the intricate genotype-phenotype relationships that underlie the clinical heterogeneity of CSF1R-RD.

In summary, CSF1R-MT induces multilevel neuronal dysfunction, combining intrinsic excitability defects with synaptic transmission failures. This dual-hit mechanism—affecting both neurons and microglia—provides a plausible explanation for the cognitive decline in CSF1R-RD patients. The 2D and 3D patient-derived organoid models recapitulate key aspects of CSF1R-RD pathogenesis and serve as valuable platforms for drug screening. It should be acknowledged, however, that these organoid models lack integrated vasculature and peripheral immune cells, which may limit their capacity to fully capture the complex neuro-immune interactions occurring in vivo. Future studies employing vascularized organoids [71] or microglia-containing assembloids, complemented by the investigation of biallelic CSF1R mutant models to delineate the specific mechanisms of neurodevelopmental forms of CSF1R-related disorders, will be essential to further elucidate the complex pathology. Despite these limitations, our findings underscore the intricate interplay between microglia and neurons in CSF1R-RD, providing important insights into potential therapeutic strategies.

## Conclusions

In this study, we present extensive evidence elucidating the pathogenic mechanisms underlying the CSF1R-MT in CSF1R-RD. First, we demonstrated that the newly identified T567M mutation induces functional impairment of CSF1R, characterized by compromised autophosphorylation at Tyr546. Given the pivotal role of microglia in CSF1R-RD pathology, we established an in vitro model utilizing iPSC-derived microglia. Our findings indicate that microglial activation occurs concomitantly with altered phagocytosis and impaired migratory capability. Transcriptomic analysis via bulk RNA-sequencing indicates that dysfunctional microglia dysregulate synaptic function pathways, suggesting a potential link to the cognitive deficits observed in CSF1R-RD. Moreover, our investigations uncover detrimental effects of the CSF1R-MT on neurogenesis and neural maturation, alongside electrophysiological abnormalities. Together, this study introduces human-derived 2D and 3D models to elucidate the pathogenic mechanisms underlying CSF1R-MT in CSF1R-RD, offering a robust platform for drug screening endeavors to develop personalized therapies for CSF1R-RD.

## Materials and methods

### Patient samples

Peripheral blood samples were obtained from clinically diagnosed CSF1R-RD patients at the National Neuroscience Institute of Singapore under SingHealth Centralised Institutional Review Board approval (2017/2602), with informed consent. For genetic validation, RNA was isolated from blood samples and reverse-transcribed using the iScript cDNA Synthesis kit (BIO-RAD, #170-8891). A sense primer on exon 11 (5′-CTGCTCCTGCTGCTATTGTACA-3′) and an antisense primer on exon 14 (5′-CAGGTGGCTCATGATCTTCA-3′) were used to amplify the mutated region. Sanger sequencing was performed to confirm the mRNA transcript of the CSF1R mutation.

### Reprogramming of PBMCs to iPSCs

The patient-derived iPSC line was generated from PBMCs of a single individual harboring the heterozygous CSF1R-T567M mutation. PBMCs were reprogrammed using the Cytotune™-iPS 2.0 Sendai Reprogramming Kit (Thermo Fisher Scientific, #A16517). The isogenic control iPSC line was subsequently generated from this patient line by CRISPR/Cas9-mediated correction of the T567M mutation locus. Both the patient-derived and isogenic control iPSCs were maintained on Matrigel (Corning, #354237)-coated dishes in mTeSR1 medium (Stemcell Technologies, #5872), with the medium being changed every other day.

### Differentiation of iPSCs to neurons

iPSCs were dissociated with Accutase (Stemcell Technology, #07922) and seeded onto ultra-low attachment culture wells with mTeSR medium for 48 h. The medium was changed to DUAL SMAD medium supplemented with Y-27632 for NPC differentiation. The DUAL SMAD medium consisted of NEAA, GlutaMAX, N2, B27, LDN, and SB431542 and was changed every other day. On day 5, the formed embryoid bodies (EBs) were seeded onto Matrigel-coated wells, and the medium was changed daily. On day 11, the attached EBs were dissociated using Accutase. The cells were then seeded onto Matrigel-coated wells with a differentiation medium. The differentiation medium, consisting of NEAA, GlutaMAX, B27, BDNF, GDNF, ascorbic acid, and dbcAMP, was changed daily for 7 days. Neurons were recorded using an Olympus FV3000 confocal microscope, and the morphology was compared using ImageJ with Sholl analysis.

### Differentiation of iPSCs to microglia

The methodology for deriving microglia from stem cells has been successfully established and applied to the study of CSF1R-RD [34, 36], providing a validated and physiologically relevant context for our functional analyses. First, iPSCs were differentiated into CD43^+^ hematopoietic progenitor cells (HPCs) using the STEMdiff™ Hematopoietic Kit (Stemcell Technology, Catalog #05310) in 12 days. Next, HPCs were differentiated into microglia using the STEMdiff™ Microglia Differentiation Kit (Stemcell Technology, #100-0019). Complete differentiation medium was supplemented with 1 mL per well every other day. After 24 days of microglial differentiation, microglia were cultured in the maturation medium (STEMdiff™ Microglia Maturation Kit, #100-0020) for an additional 4 days before use in studies. Microglia were recorded using an Olympus FV3000 confocal microscope, and the morphology was compared using ImageJ with Skeleton analysis [72].

### Differentiation of iPSCs to COs

iPSCs were dissociated with Accutase and seeded onto 96-well round-bottom ultra-low attachment plates with EB Seeding Medium for 48 h. On day 2 and day 4, part of the medium was removed, and the fresh EB Seeding Medium was added into the culture plates. On day 5, the formed EBs were transferred to 24-well ultra-low attachment plates. On day 7, the expansion medium (STEMdiff™ Cerebral Organoid Basal Medium 2 + Supplement C and D) was prepared. In a 50 mL conical tube, 200 μL of liquid Matrigel was added to 10 mL of ice-cold expansion medium, and the EBs were transferred to 6 wells with the expansion medium. The plates were placed on an orbital shaker and incubated at 37 °C, 5% CO_2_ for 3 days. On day 10, the medium was changed into cerebral organoid maturation medium (STEMdiff™ Cerebral Organoid Basal Medium 2 + Supplement E) and changed every 3 days. Images of tissue cultures were captured using an Olympus DP73 microscope.

### Co-culture of microglia with COs

5 × 10^5^ iPSC-derived microglia were collected and co-cultured with each day-60 organoid. The co-culture plates were cultured in a 37 °C incubator with 5% CO_2_. The co-culture system was maintained in Cerebral Organoid Maturation Medium, and the medium was changed every other day.

### Whole-cell patch-clamp recordings

On day 130 (D130) of the organoids, we performed whole-cell patch-clamp recordings to analyze the electrical properties of the neurons. The internal solution contained 130 mM K-gluconate, 10 mM KCl, 5 mM EGTA, 10 mM HEPES, 1 mM MgCl_2_, 0.5 mM Na_3_GTP, 4 mM Mg-ATP and 10 mM Na-phosphocreatine. pH was adjusted to 7.4 with KOH. The external solution contained 10 mM glucose, 125 mM NaCl, 25 mM NaHCO_3_, 1.25 mM NaH_2_PO_4_.2H_2_O, 2.5 mM KCl, 1.8 mM CaCl_2_, and 1 mM MgCl_2_. The whole-cell recording was performed with a Multi-clamp 700b amplifier (Molecular Device), low-pass filtered at 1 kHz, and the series resistance was typically <20 MΩ after >50% compensation. The P/4 protocol was used to subtract the leakage and capacitive transients online. In the process of detecting voltage-gated currents, neurons were held at a potential of −70 mV, then the voltage-gated current was evoked with different test potentials ranging from −90 to 50 mV in 10 mV increments. The profiles of the peak inward sodium current and outward potassium currents of the respective types of correction and CSF1R-T567M mutant COs were recorded. To detect the action potential firing, neurons were held at 0 pA current and then injected with increasingly positive currents ranging from −20 to 40 pA in 5 pA increments. The amplitude and frequency of spontaneous excitatory postsynaptic current and spontaneous inhibitory postsynaptic current were recorded with the voltage held at −70 mV.

### Autophosphorylation assay

SH-SY5Y CSF1R-WT and CSF1R-MT stable-expressing cells were seeded into 6-well plates and were incubated at 37 °C overnight. The Dulbecco’s modified Eagle medium (DMEM) culture medium was replaced with a serum-free medium for 24 h. The cells were treated with 50 ng/mL of CSF1 (Sigma-Aldrich, #M9170) for 0, 5, 15, and 40 min. The cells were collected for immuno-blotting after washing with PBS.

### Cytokine assay

To evaluate cytokines secreted by microglia in vitro, we collected conditioned cell medium to perform an assay. 1 × 10^5^ microglia were treated with 100 ng/mL lipopolysaccharide (LPS) (Sigma-Aldrich) for 24 h. Medium without LPS was used as a control. The culture supernatant was added to a prepared membrane according to the manufacturer’s instructions (RnD Systems, ARY005B). The nitrocellulose membranes contained 36 different capture antibodies printed in duplicate. After incubation, the membranes were added with the Chemi Reagent Mix evenly and exposed to an autoradiography film processor.

### Bulk RNA-sequencing

Patient iPSC-derived microglia were washed with PBS. Total RNA was prepared using the Qiagen RNeasy Mini Kit (Catalog #05310) according to the standard protocol. Suitable samples were used for library construction. The libraries were subjected to sequencing on an Illumina platform, and paired-end reads were generated. Raw reads in fastq format were first processed using FASTQ processing tools. All the downstream analyses were based on clean, high-quality data. FeatureCounts v1.5.0-p3 was used to count the number of reads mapped to each gene. Before differential gene expression analysis, the read counts for each sequenced library were adjusted by a scaling normalization factor using the edgeR program package. Differential expression analysis of the two groups was performed using the edgeR R package (3.22.5). Gene Ontology (GO) enrichment analysis of differentially expressed genes was performed using the clusterProfiler R package, in which gene length bias was corrected. The clusterProfiler R package was used to test the statistical enrichment of differentially expressed genes in KEGG pathways. For visualization in figures, the upregulated and downregulated genes were selected based on the combined ranking of adjusted p-value and magnitude of log2FoldChange.

### Migration assay

To assess the migratory capacity of microglia, 5.5 × 10^4^ microglia were plated in the upper chamber of trans-well plates (5-µm-pore polycarbonate filters in 24-well, Corning) and adenosine triphosphate (ATP, 100 µM; Epicentre) was added in the lower chamber. Cells without ATP treatment were used as controls. After 4 h, the cells were fixed in 4% paraformaldehyde for 15 min at room temperature. Cells at the top surface of the chamber were removed with a cotton swab. Cells at the bottom side of the chamber were stained with Hoechst for 10 min to visualize cell nuclei.

### Phagocytosis assay

Coverslips were coated with 10 μg/mL poly-D-lysine at 4 °C overnight, then washed with distilled water 3 times. The poly-D-lysine-coated coverslips were then coated with 20 μg/mL laminin in PBS for 48 h in the incubator. Purified myelin was first isolated from adult mouse brains via sucrose gradient ultracentrifugation and labeled with pHrodo (Thermo Fisher) prior to use [73]. To evaluate the phagocytic capacity of microglia, 5 × 10^4^ microglia were seeded onto coverslips and cultured in an incubator containing 5% CO_2_ and 100% humidity at 37 °C. After attachment, microglia were incubated with 0.01% (v/v) fluorescent latex beads (1 μm diameter, Sigma-Aldrich, #L1030) or the pHrodo-labeled myelin. The coverslips were then washed with PBS and fixed with 4% paraformaldehyde for 15 min. Microglia were immunostained with IBA1 (FujiFilm Wako, #PTN5930) and recorded using a confocal microscope. Images were analyzed using ImageJ software.

### Generation of stable cell lines

pEF6-CSF1R-WT plasmid containing the full-length murine CSF1R coding sequence was a gift from Dr. Clare Pridans, University of Edinburgh. Site-directed mutagenesis using primer pair 5′ ATCGACCCCATGCAGCTGCCTTACAACGAGAAGT 3′ and 5′ AGGCAGCTGCATGGGGTCGATGAAAGTATAACTGTTGCCC 3′ was carried out to mutate C1700T of the CSF1R coding sequence, resulting in a threonine to methionine conversion at position 567 of the CSF1R amino acid sequence. The PCR product was then heat-shocked into E*. coli* DH5α competent cells and grown on a Luria-Bertani agar plate containing 100 mg/ml carbenicillin after clean-up. Plasmids were extracted from the forming colonies. 2.5 μg of pEF6-CSF1R-WT and pEF6-CSF1R-MT plasmids were transfected into SH-SY5Y, BV2 cells, and human microglial HMC3 (ATCC, #CRL-3304) in a 6-well plate using Lipofectamine® LTX transfection reagent (Invitrogen, #15338100) according to the manufacturer’s instructions [74]. Two days after transfection, 20 μg/mL blasticidin (InvivoGen, #ant-bl-05) was used to select stable cell lines. The stable cell lines were maintained in DMEM (Sigma-Aldrich, #D1152) culture medium without blasticidin.

### Western Blotting

Western blotting assays were performed as previously described [75, 76]. The antibodies used in this study were CSF1R (Cell Signaling, #3152), phospho-CSF1R Tyr708 (Cell Signaling, #3080), phospho-CSF1R Tyr723 (Cell Signaling, #3151), phospho-CSF1R Tyr546 (Cell Signaling, #3083), β-actin (Santa Cruz, #AC-15), LC3 (Abcam, #ab243506), IBA1 (Fujifilm Wako, #019-19741), and TUJ1 (Millipore, #MAB1637). On the second day, the membranes were incubated with secondary antibodies conjugated with horseradish peroxidase against mouse or rabbit IgG (GE Healthcare, #NA931V and #NA934V) for 1 h at room temperature. Autoradiography was performed using enhanced chemiluminescence substrate or West Femto substrate (Thermo Fisher Scientific, #32019 and #34096) and developed by a ChemiDoc MP imaging system (Bio-Rad). Signal intensities of immunoblots were quantified using ImageJ.

### Quantitative RT-PCR

For RT-PCR, 1 µg of total RNA was converted to complementary DNA (cDNA) using the iScript cDNA Synthesis kit (Bio-Rad, #170-8891). qPCR was conducted in a LightCycler system (Roche) with the 2x All-in-One qPCR reaction mix (GeneCopoeia, #QP001-01). β-actin was used as the internal control. Relative quantification was performed using the 2^−ΔΔCt^ method.

### Immunohistochemistry staining

Cryosections were washed with 1 × PBS and permeabilized with 0.1% Triton X-100 and blocked with 1% BSA for 1 h at room temperature. Then the samples were incubated with primary antibodies overnight at 4 °C. Primary antibodies included TMEM119 (Cell Signaling, #41134), IBA1 (FujiFilm Wako, #PTN5930), P2RY12 (Invitrogen, # 702516), KI67 (Abcam, #ab15580), TUJ1 (Novus, #NB100-1612), Cleaved Caspase-3 (Cell Signaling, #9661), MAP2 (Novus, #NB300-213), PSD95 (Abcam, #ab18258) and Synaptophysin (Abcam, #ab52636). On the second day, samples were incubated with secondary antibodies and DAPI (4′,6-diamidino-2-phenylindole) for 1 h at room temperature. Slides were sealed, and images were captured using an Olympus FV3000 confocal microscope.

### Statistical analyses

For each experiment, at least three independent measurements were performed. All statistical analyses were performed using GraphPad Prism 8 software. Data are presented as mean ± SD for all statistical analyses. Two-tailed Student’s *t*-test was used to compare the differences between the two groups. Two-way ANOVA with Tukey’s *post hoc* test was used for the comparison of multiple groups. The statistical significance levels were set at *p < 0.05, **p < 0.01, and ***p < 0.001.

### Ethics approval and consent to participate

Informed consent was obtained from patients. The use of human samples in this study was approved by SingHealth Centralised Institutional Review Board (2017/2602). All methods were performed in accordance with the relevant guidelines and regulations.

## Supplementary information

Supplementary Materials

## Data Availability

All data generated or analyzed during this study are included in this article and the Supplementary Materials.
