# Supplementary material for: CSF1R T567M mutation induces microglial dysfunction and synaptic impairment in patient iPSC-derived cerebral organoids of CSF1R-related disorder
Source: Cell Death Discov. 2026 Mar 12;12:148. doi: 10.1038/s41420-026-02995-2 (PMC13039969; doi:10.1038/s41420-026-02995-2)
Supplement: Supplementary file 1 — Supplementary Materials [file 41420_2026_2995_MOESM1_ESM.docx]

**Supplementary Materials for**

**CSF1R T567M mutation induces microglial dysfunction and synaptic impairment in patient iPSC-derived cerebral organoids of CSF1R-related disorder**

**Li CHI^1,2, #^, Haitao TU^2,9 #^, Zhihong LI^2,9^, Lifeng QIU^2^, Zhi-Wei ZHANG^2^, Sook-Yoong CHIA^2^, Jayne Yi TAN^3^, Ivy A.W. HO^4,5,6^, Yuin-Han LOH^7^, Eng-King TAN^3,8,9^, Wei TENG^1^, Zhong PEI^10^, Zbigniew K. WSZOLEK^11^, Adeline S.L. NG^3,9^, Li ZENG^2,9,12, *^**

^1^Hospital of Stomatology, Guangdong Provincial Key Laboratory of Stomatology, Institute of Stomatological Research, Guanghua School of Stomatology, Sun Yat-sen University, Guangzhou, 510055, China.

^2^Neural Stem Cell Research Lab, Research Department, National Neuroscience Institute, Singapore, 308433, Singapore.

^3^Department of Neurology, National Neuroscience Institute, Singapore, 308433, Singapore.

^4^Molecular Neurotherapeutics Laboratory, National Neuroscience Institute, Singapore, 308433, Singapore.

^5^Department of Physiology, Yong Loo Lin School of Medicine, National University of Singapore, Singapore, 119228, Singapore

^6^Duke-NUS Medical School, Singapore, 169857, Singapore

^7^Institute of Molecular and Cell Biology (IMCB), A*STAR (Agency for Science, Technology and Research), Singapore, 138673, Singapore

^8^Research Department, National Neuroscience Institute, Singapore General Hospital (SGH) Campus, Singapore, 169856, Singapore

^9^Neuroscience & Behavioral Disorders Program, DUKE-NUS Graduate Medical School, Singapore, 169857, Singapore

^10^Department of Neurology, The First Affiliated Hospital, Guangdong Provincial Key Laboratory of Diagnosis and Treatment of Major Neurological Diseases; National Key Clinical Department and Key Discipline of Neurology, Sun Yat-Sen University, Guangzhou, 510080, China.

^11^Department of Neurology, Mayo Clinic Florida, Jacksonville, Florida 32224, USA

^12^Centre for Molecular Neuropathology, Lee Kong Chian School of Medicine, Nanyang Technological University, Novena Campus, 11 Mandalay Road, Singapore, 308232, Singapore.

^#^ These authors contributed equally to this work.

^*^Corresponding author: Li ZENG, PhD, Email: Li_Zeng@nni.com.sg

**Supplementary Fig. S1** (Original western blot images in **Fig. 1**)


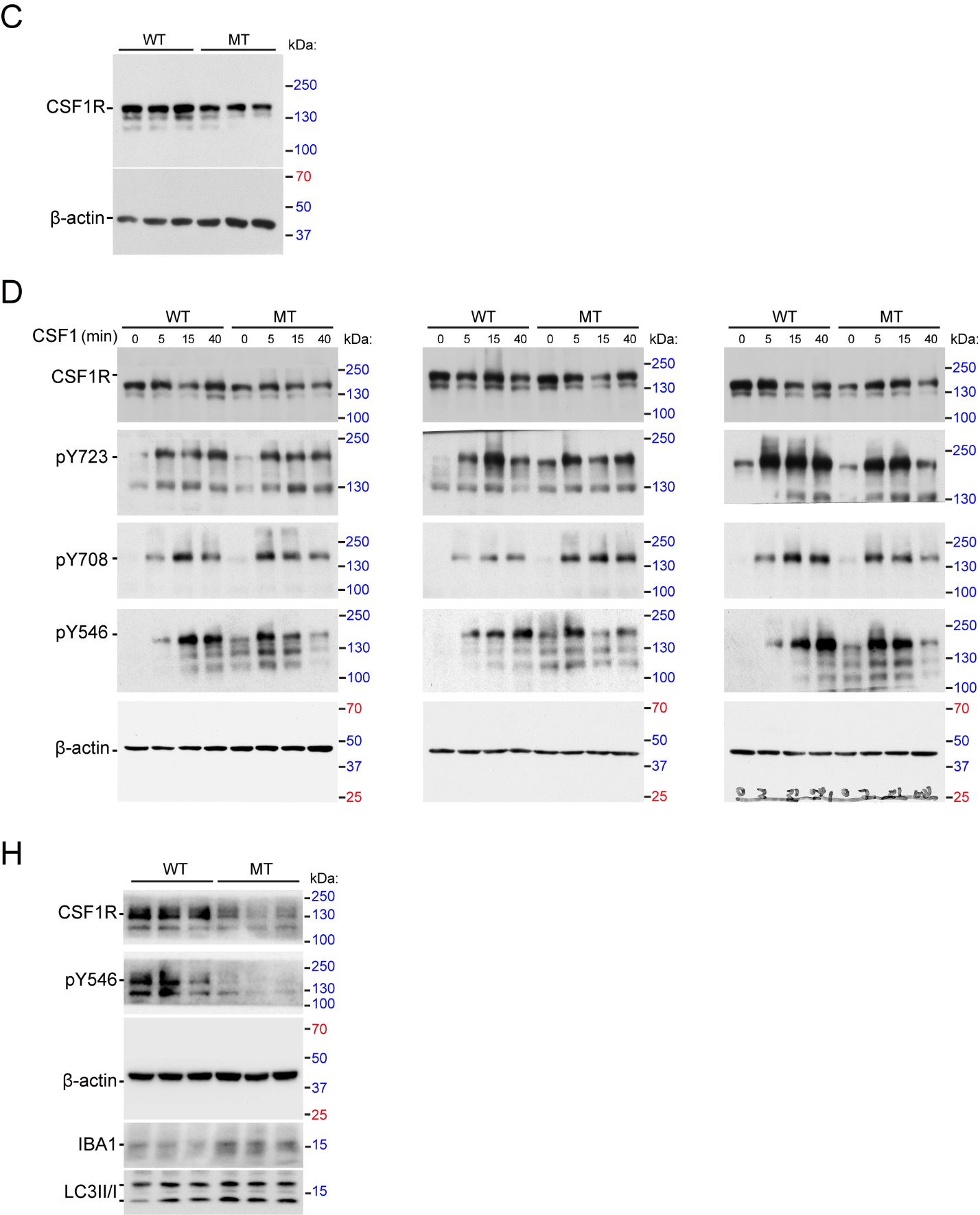


**Supplementary Fig. S2**


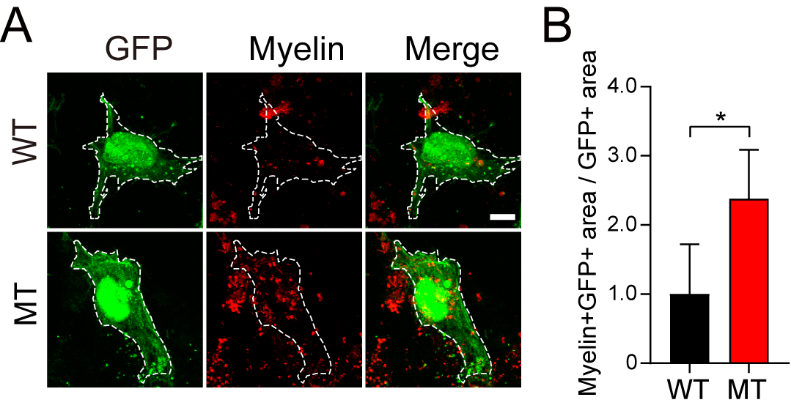


**Supplementary Fig. S2. CSF1R-MT enhances phagocytosis of myelin debris in HMC3 cells. (A)** Representative images of HMC3 cells (green) after a 6-hour incubation with pHrodo Red-myelin (red). Scale bar = 10 µm. **(B)** Statistical analysis of the phagocytic ability of the pHrodo-labeled myelin. Fields of view: Ctrl =7, MT=4. Data presented as Mean ± SD. Two-tailed Student’s *t*-test was used to compare the differences between the two groups. The statistical significance levels were set at **p* < 0.05.

**Supplementary Fig. S3** (Original western blot images in Fig. 4)


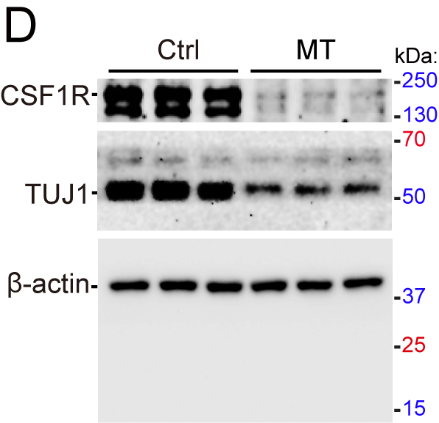


**Supplementary Fig. S4**


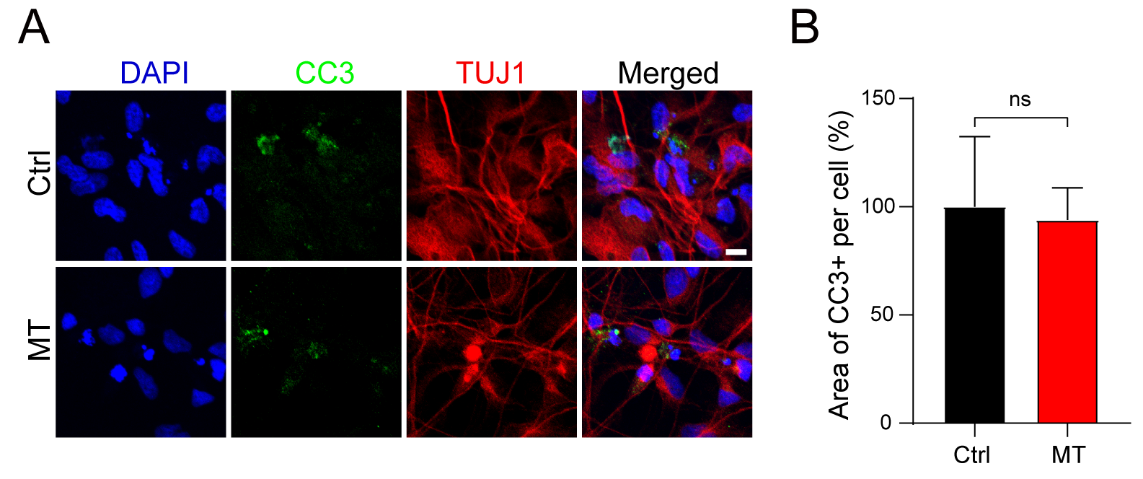


**Supplementary Fig. S4. CSF1R-MT does not alter apoptosis in iPSC-derived neurons. (A)** Representative images of control (Ctrl) and mutant (CSF1R-MT) neurons stained for the apoptosis marker Cleaved Caspase-3 (CC3, green) and the neuronal marker TUJ1 (red). Nuclei are counterstained with DAPI (blue). Scale bar = 10 µm. Data presented as Mean ± SEM. Fields of view: Ctrl = 7, MT = 10. Two-tailed Student’s *t*-test was used to compare the differences between the two groups. The statistical significance levels were set at **p* < 0.05.
